# Supplementary material for: Comparative multiomics analysis of cell physiological state after culture in a basket bioreactor
Source: Sci Rep. 2022 Nov 23;12:20161. doi: 10.1038/s41598-022-24687-4 (PMC9686226; doi:10.1038/s41598-022-24687-4)
Supplement: Supplementary file 1 — Supplementary Information 1. [file 41598_2022_24687_MOESM1_ESM.zip › raw data/Proteomic raw data/1.data_control/MW_distri.pdf]

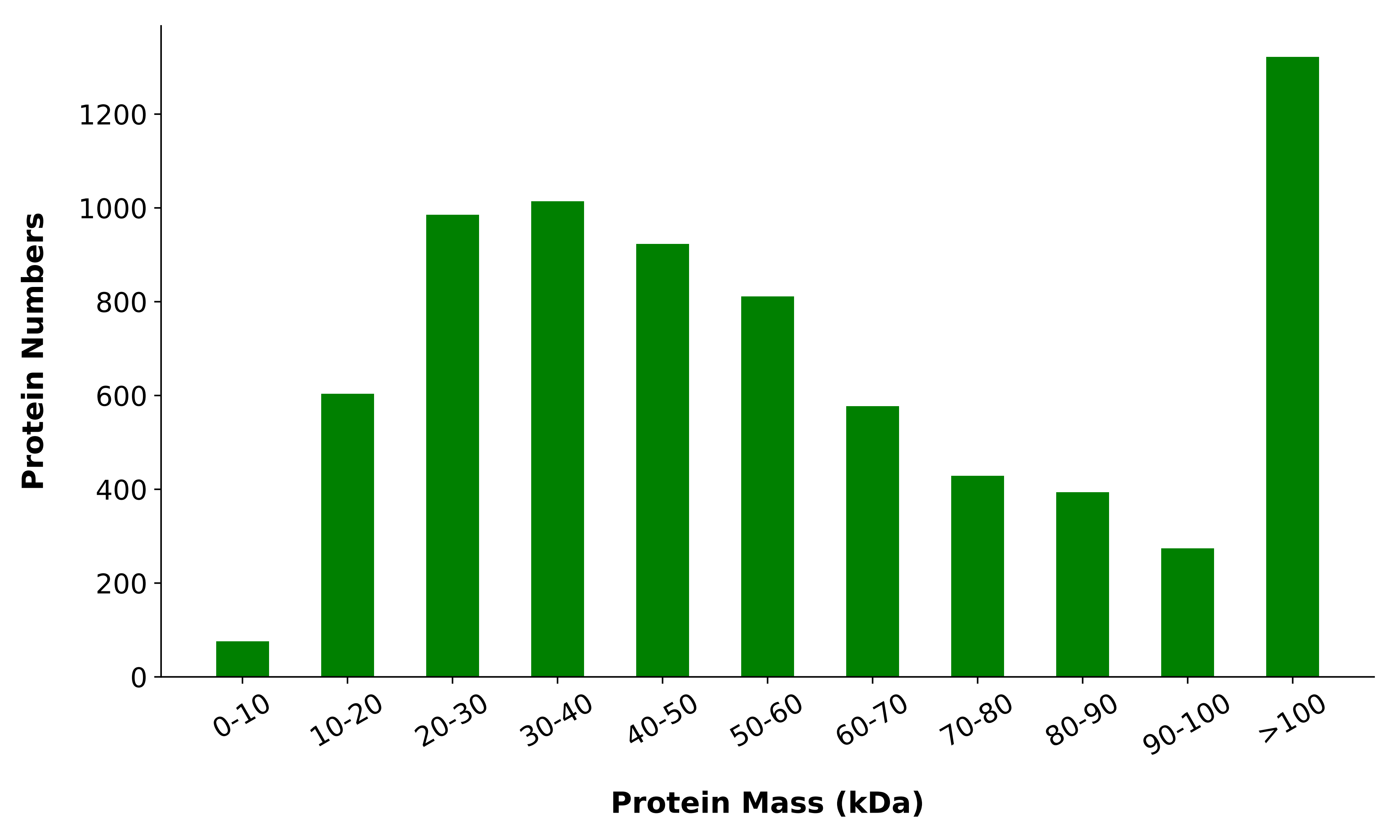
Protein Numbers
0
200
400
600
800
1000
1200
0-10
10-20
20-30
30-40
40-50
50-60
60-70
70-80
80-90
90-100
>100
Protein Mass (kDa)
